# The Role of Phytohormones in Mediating Drought Stress Responses in *Populus* Species

**DOI:** 10.3390/ijms26083884

**Published:** 2025-04-19

**Authors:** Sajid Ali, Sana Tahir, Syed Shaheer Hassan, Meiqi Lu, Xinyu Wang, Lai Thi Quynh Quyen, Wenbo Zhang, Su Chen

**Affiliations:** 1 State Key Laboratory of Tree Genetics and Breeding, Northeast Forestry University, Hexing Road, Harbin 150040, China; sajidaliaup@gmail.com (S.A.); tsana011@gmail.com (S.T.); wangxinyu990927@163.com (X.W.);; 2 Heilongjiang Province Key Laboratory of Sustainable Forest Ecosystem Management-Ministry of Education, School of Forestry, Northeast Forestry University, Harbin 150040, China; shaheerhassan372@gmail.com; 3 International Center for Bamboo and Rattan, No. 8, Futong Eastern Avenue, Wangjing Area, Chaoyang District, Beijing 100102, China

**Keywords:** phytohormones, root development, endophytic colonization, nutrient uptake, *Populus*

## Abstract

Drought stress substantially impacts the development and viability of *Populus* spp., which are essential for forestry and bioenergy production. This review summarizes and describes the functions of phytohormones, such as abscisic acid, auxins, and ethylene, in modulating physiological and molecular responses to water scarcity. Drought-induced ABA-mediated stomatal closure and root extension are essential adaptation processes. Furthermore, auxin–ABA (abscisic acid) interactions augment root flexibility, whereas ethylene regulates antioxidant defenses to alleviate oxidative stress. The advantageous function of endophytic bacteria, specifically plant growth-promoting rhizobacteria (PGPR), can augment drought resistance in spruce trees by enhancing nutrient absorption and stimulating root development. Structural adaptations encompass modifications in root architecture, including enhanced root length and density, which augment water uptake efficiency. Similarly, Arbuscular Mycorrhizal Fungi (AMF) significantly enhance stress resilience in forest trees. AMF establishes symbiotic relationships with plant roots, improving water and nutrient uptake, particularly phosphorus, during drought conditions. Furthermore, morphological alterations at the root–soil interface enhance interaction with soil moisture reserves. This review examines the complex mechanisms by which these hormones influence plant responses to water shortage, aiming to offer insights into prospective techniques for improving drought tolerance in common tree species and highlights the importance of hormone control in influencing the adaptive responses of prominent trees to drought stress, providing significant implications for research and practical applications in sustainable forestry and agriculture. These findings lay the groundwork for improving drought tolerance in *Populus* spp. by biotechnological means and by illuminating the complex hormonal networks that confer drought resistance.

## 1. Introduction

Everywhere across the globe, drought is a significant abiotic stressor that has a detrimental impact on the development, productivity, and survival of plants [1]. The reduction in stomatal transpiration, the enhancement of the roots’ capacity to absorb water, the preservation of cellular water content, and the activation of the antioxidant system in order to maintain redox homeostasis are the mechanisms by which plants compensate for the effects of drought stress [2]. Cold, drought, extreme temperatures, salinity, and pathogen invasion increase reactive oxygen species (ROS), causing oxidative stress. Oxidative stresses damage proteins, lipids, and DNA, limiting respiration and photosynthesis [3]. Severe drought stress, the primary abiotic factor affecting *Populus* growth, can significantly impede its growth, development, and reproductive capacity [4,5]. The *Populus* forest covers about 8 million hectares (ha), which is more than 14% of China’s planted forest area, and has a total carbon store of around 261 metric tons (Tg), according to the country’s ninth national forest inventory [6]. In the genus *Populus*, approximately 30 different types of trees are collectively referred to as “poplars”. The trees are usually categorized into six groups: *Populus*, *Tacamahaca*, *Leucoides*, *Turanga*, *Abaso*, and *Aigeiros* [7]. The genus represents one of the most complex groups in plant taxonomy, partly because of the frequent occurrence of natural hybridization [8]. *Populus* species are most diverse in the US and China, where the former classifies 29 species and 27 hybrids [9]. Consequently, it is imperative to identify innovative drought-resistant genes, elucidate their regulatory processes, and develop new drought-resistant types of *Populus* spp. [10]. Gibberellin, auxin, cytokinin, ethylene, strigolactone, jasmonic acid, salicylic acid, and brassinosteroid are the eight known phytohormones that trigger a chain reaction of responses to stimuli in the environment [11]. Additionally, antioxidant enzyme overexpression during drought stress can reduce reactive oxygen species (ROS)-induced oxidative damage [12]. An essential intercellular signal molecule, hydrogen peroxide (H_2_O_2_), is a reactive oxygen species (ROS) generated by plant cells metabolic processes. H_2_O_2_ regulates a number of downstream gene expression changes through its interactions with a number of plant hormones and signaling pathways, including salicylic acid (SA), jasmonic acid (JA), auxin, ethylene (ETH), and abscisic acid (ABA) [13]. With their role as chemical messengers synthesized and transported throughout the plant, these hormones regulate a wide range of physiological and developmental processes [14]. Of particular note, the abundance of JA and SA hormones in higher plant species has focused research on plant response and defense mechanisms [15]. Phytohormones and reactive oxygen species (ROS) are intricately interdependent, with the former regulating the latter’s synthesis [16].

To better understand how various phytohormones, such as cytokinin, auxins, and abscisic acid, regulate the biochemical and physiological responses of poplar trees to drought-induced stress, this review will examine the literature on the topic. Molecular methods of interaction with stress-responsive genes and signaling cascades. The effects of soil moisture and microbial interactions on phytohormonal control are likewise unclear. To address these gaps, we analyze the functions of principal phytohormones in regulating biochemical and physiological responses of poplar trees during drought stress. We examined the molecular processes by which phytohormones modulate stress-responsive gene expression. We also examined the interplay of phytohormones, soil moisture, and microbial populations in improving drought resilience and obtained insights into the intricate hormonal signaling networks, establishing a basis for biotechnological approaches to enhance the drought resilience of *Populus* spp.

## 2. Harmful Effects of Drought Stress on Plants

Drought stress has a profound impact on agricultural productivity, particularly by reducing crop yield and compromising grain quality [17,18]. It is widely regarded as one of the most limiting factors in modern agriculture [18]. Drought conditions during key developmental stages such as flowering and grain filling can drastically reduce grain size and number, leading to significant yield losses in staple crops like wheat and maize [19,20]. Moreover, insufficient soil moisture affects nutrient uptake, further stunting plant growth and reducing overall crop performance. The combined effect of climate change and frequent drought events has already led to declining agricultural productivity, which is expected to worsen in the coming years [21,22,23]. This intensifies concerns about food insecurity, particularly in vulnerable regions affected by erratic rainfall patterns and extended dry seasons [24].

In addition to yield losses, drought also affects the nutritional and health value of crops. Water stress induces a shift in metabolic pathways and triggers complex defense mechanisms, altering the biosynthesis of key metabolites and nutritional compounds [25]. As a result, the quality of produce—such as protein content, oil composition, and micronutrient levels—may be negatively affected, further diminishing the value of agricultural outputs.

At the physiological level, drought leads to a reduction in soil water content (SWC), which in turn lowers hydraulic conductivity from the roots to the leaves. Plants respond by closing their stomata to conserve water, especially under conditions of high vapor pressure deficit (VPD). While this response limits water loss, it also restricts carbon dioxide intake, resulting in reduced CO_2_ fixation and photosynthesis [26]. Prolonged stomatal closure impairs carbohydrate production and causes a carbon imbalance, thereby affecting energy availability for growth and development.

Water deficit also causes osmotic stress due to increased solute concentration in cells, reducing turgor pressure and impairing cell expansion and organ development. The stalling of the photosynthetic electron transport chain results in the production of ROS which affects various macromolecules [20]. Consequently, drought stress often leads to the overproduction of reactive oxygen species (ROS), which damage proteins, lipids, and cellular membranes. Xylem cavitation and embolism become more likely under low soil water potential, which may result in partial or complete hydraulic failure and even plant death [27,28].

Reactive oxygen species (ROS) are generated through several metabolic pathways located in different cellular compartments. In the chloroplasts, ROS, such as singlet oxygen and superoxide, are produced during photosynthesis, particularly under high light intensity or drought conditions that limit CO_2_ availability. In the mitochondria, the electron transport chain leaks electrons during respiration, forming superoxide radicals, especially under stress conditions that disrupt ATP synthesis. In the peroxisomes, photorespiration leads to hydrogen peroxide (H_2_O_2_) accumulation, while in the apoplast, ROS are produced by NADPH oxidases during pathogen attack or abiotic stress [29]. To mitigate ROS-induced damage, plants activate a range of detoxification mechanisms. These include enzymatic antioxidants like superoxide dismutase (SOD), ascorbate peroxidase (APX), catalase (CAT), glutathione peroxidase (GPX), and peroxiredoxin (PRX), as well as non-enzymatic antioxidants like ascorbate and glutathione (GSH) [30]. Chloroplasts act as environmental sensors; their photosynthetic apparatus is known to be affected by the number of biotic (bacteria, viruses, etc.) and abiotic (heat, strong light, salt, hypoxia, etc.) stresses. Apart from assaying ROS production, another indicator of the damage to photosystems is decreased chlorophyll fluorescence [31]. The levels of antioxidant enzyme activity and chlorophyll fluorescence are impacted by drought [32].

**Figure 1:**
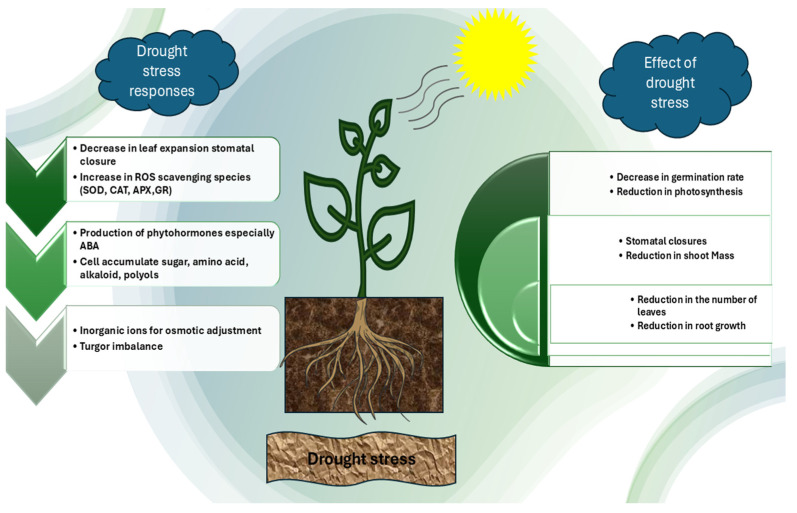
In response to water scarcity, plants close their stomata, which reduces transpiration and photosynthesis. As a result, plants may experience stunted growth, withering, or even death in extreme circumstances due to their inability to absorb enough water.

These defense systems, although critical, are often overwhelmed under severe drought conditions, leading to oxidative stress and cellular injury. An overview of these harmful effects is illustrated in Figure 1.

## 3. Drought Stress and Hormonal Influences on Root–Soil Interface Adaptations

Hormones trigger these changes. Stomatal closure and root development depend on abscisic acid (ABA). Drought resilience depends on hormone balance, including cytokinins and auxins, which affect root structure [33]. The *PtrABR1* gene in *Populus trichocarpa* is significantly upregulated by drought stress, facilitating lateral root development and improving drought resilience. This indicates that hormonal mechanisms, including abscisic acid (ABA) and other hormones, are essential in governing root responses during water deficiency circumstances [34]. Drought-tolerant *Populus* species have an increased fine root density and root architecture to facilitate water absorption. Water retention under low-moisture conditions is helped by specialized tissues such as thicker rhizodermis [35]. Fine roots are primarily responsible for completing nitrogen (N) and carbon (C) cycles, as well as absorbing large amounts of water and nutrients, because of their short lifespan and quick turnover rate [36,37,38]. This ensures that there will be enough water and nutrients for plants to develop and undergo photosynthesis [38]. A root’s delicate anatomical structure is a direct reflection of its growth and development, demonstrating the intimate relationship between anatomy and physiological function [39]. Environmental factors can affect the structure of fine roots [37]. In addition to directly influencing plant roots, temperature can indirectly affect fine roots by altering the soil’s nutritional conditions [40]. Spreading roots that are deeper assists plants absorb more water and nutrients in colder climates. Also, high temperatures accelerate fine root development [41,42]. The response of fine roots to drought is influenced by the intricate interplay between anatomical structure and function [43]. Without water, hydraulic conductivity, root length, rhizosphere wettability, soil structure, and porosity vary. In the rhizosphere, plants deposit organic substances. This influences chemical, biological, and physical rhizospheres. Mucilage, secretions, root exudates, sloughed-off cells, dead root cells, and root enzymes exhibit distinct properties when moisture levels are low in rhizodeposits [44]. Root exudates contain mostly proteins and mucilage, with low-molecular-weight carbohydrates, amino acids, flavonoids, coumarin, and carboxylates. Multiple-purpose root exudates alter stress tolerance [45]. Due to limited carbon transfer from plants to bacteria and non-mycorrhizal fungi in the rhizosphere, droughts impede plant–microorganism interactions [46,47,48]. Studies indicate that grapevines exhibiting elevated levels of suberin in their roots demonstrate enhanced resilience to drought conditions [49]. However, a root may become more susceptible to damage if suberin accumulates in its fine roots. Research has also shown that lignin deposition can enhance drought tolerance by forming an impermeable water-resistant barrier around xylem tissue [50]. The rhizosphere microbiome is the bacterial community inside a few millimeters of plant roots [51]. Comprehending the molecular mechanisms behind these changes is crucial for enhancing drought tolerance in *Populus* species. Research indicates that altering hormonal pathways may improve root resistance and overall plant performance during drought stress [52].

**Figure 2:**
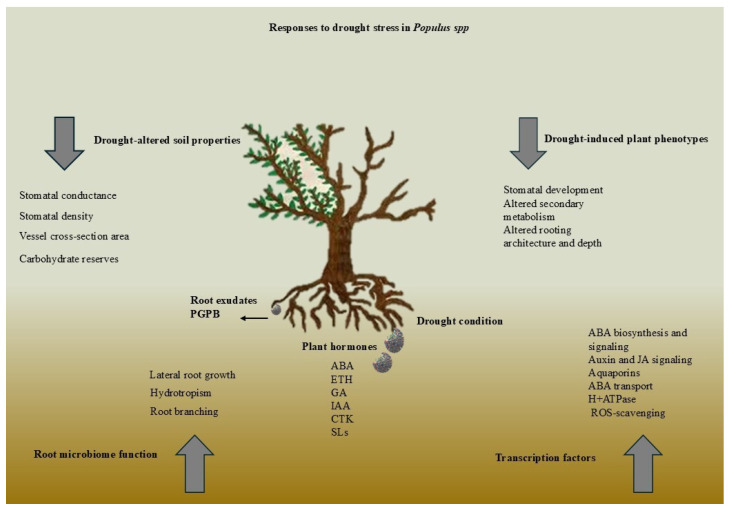
Hormonal networks significantly influence the adaptations of plants at the root–soil interface under drought conditions by modulating root architecture, regulating water uptake mechanisms, and integrating complex signaling pathways that enhance resilience against water stress.

Drought stress regulates root architecture, water uptake, and stress resilience through complex hormonal signaling at the root–soil interface. Auxins, cytokinins, and ethylene regulate root elongation and branching, while ABA (abscisic acid) stimulates deep root growth and stomatal closure. Figure 2 shows how hormones improve water uptake and drought adaptation.

## 4. Role of Endophytes in Drought Tolerance

Piriformospora indica colonization primarily transpires in the cortex and root epidermis of many host plants, including poplar, rice, barley, tobacco, Arabidopsis, and maize [53]. Plants experiencing abiotic stresses, including salinity, hydric stress, drought, low temperatures, and heavy metal toxicity [54] derive advantages from the support of P. indica [55]. Plants may provide microbes with 15–20% of their photosynthetic output in return for beneficial services; however, this is generally surpassed by the advantages conferred by bacteria [56]. The fungus is probably the best-recognized endophyte [57]. Plants that colonize *Piriformospora indica* symbiotically grow more quickly and are less affected by biotic stress [58]. Trichoderma spp. has been widely used in agricultural applications due to its well-known biological control mechanism [59]. Root rot disease and other prevalent plant diseases can be effectively treated by *Trichoderma* spp., according to recent research [60], via which the root architecture is well controlled and the length is increased [61]. Plant growth is influenced by a multitude of factors, including temperature, light intensity, nutrient availability, and microbial ecology. The roots release a great deal of photosynthetic byproducts, which enrich the soil in the area immediately around the roots, creating a nutrient-rich environment known as the rhizosphere [62]. Furthermore, it was identified that *T. virens* and *T. atroviride* produce compounds associated with auxin and indole acetic acid (IAA). IAA is a plant hormone of the auxin class [63].

**Figure 3:**
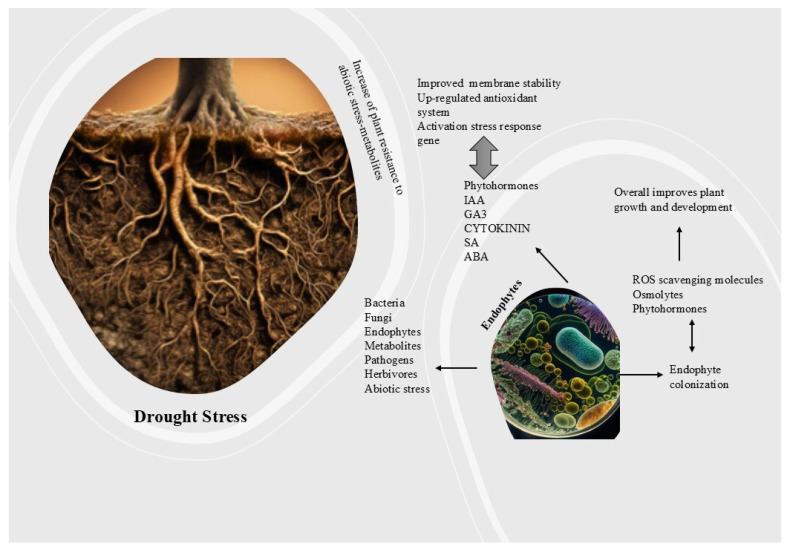
Drought-resistant endophytes offer a promising approach to improving plant resistance against climate-related water stress. These microorganisms can substantially aid sustainable agricultural practices and forest management strategies by enhancing nutrient absorption, facilitating advantageous hormonal alterations, and improving physiological responses to drought, thereby mitigating its effects on plant health and productivity.

Recent research has revealed multiple strains of drought-resistant endophytic fungus from various plant species, as illustrated in Figure 3.

## 5. Phytohormone Regulation of Root Development Under Drought

Plants produce a diverse array of phytohormones, such as auxin, cytokinin (CK), gibberellic acid (GA), ethylene, salicylic acid (SA), and jasmonic acid (JA), to facilitate plant development. These phytohormones facilitate a diverse array of dynamic yet precisely calibrated molecular responses throughout the life cycle of a plant [64]. Some of the drought-related activities that phytohormones including ethylene, salicylic acid, jasmonic acid, and abscisic acid regulate include root architectural adjustments and root cell osmotic potential preservation. Root development and growth depend on phytohormonal regulation and signaling in both ideal and poor conditions. When plants experience drought stress, they change the structure and function of their roots, and ABA (abscisic acid) is a key component in this process [65,66]. A lot of studies have focused on ABA (abscisic acid), a well-known plant hormone that suppresses development in response to stress, as a potential way to increase drought tolerance in plants [67]. Many methods have been used to isolate phytohormones to learn more about their effects. Chromatography is one of the two most used techniques for separating plant growth hormones [68,69]. Calcium enhances seedling growth with ABA (abscisic acid), IAA, and MeJA, but inhibits growth with GA3 or SA [70]. Increased respiration by the shoots is another mechanism by which drought stress keeps metabolic activity constant. Then, the glucose levels in the citrus plants’ storage organs drop [27]. Additionally, to counteract these adverse conditions, plant growth regulators are sprayed externally [71]. See Table 1 for a summary of how phytohormones improve plant growth, development, and productivity while simultaneously increasing their drought resistance.

## 6. Role of Hormones

### 6.1. Auxin

Auxin is crucial in drought conditions as it facilitates the formation of a robust root system. Indole-acetic acid (IAA), a naturally occurring auxin, is biosynthesized in plants in a tryptophan-dependent yet independent manner [81]. *PeFUS3* regulated lateral root growth during drought stress through auxin signaling. Furthermore, *PeFUS3* directly enhanced the expression of *PePYL3*, and poplar lines with overexpressed *PePYL3* demonstrated markedly improved drought resistance [82]. *PtHB180-OE* plants exhibited pleiotropic phenotypes, characterized by enhanced plant height and reduced leaf area due to auxin modulation. *PtHB180* may serve as a viable candidate gene for enhancing drought resistance through a genetic transformation in poplar [83]. The *IAA17.1/HSFA5a* module collectively regulates flavonol production and controls ROS buildup, consequently altering the root system of poplar to respond to salinity stress [84]. Auxin’s negative control of *DRO1* during drought, which in turn changes the root growth symmetry [85]. Contrarily, research into auxin’s role in drought stress has made use of *TLD1/OsGH3.13*, the gene encoding indole-3-acetic acid (IAA)–amido synthase; this, in turn, enhances plant resistance to drought stress by increasing the expression of genes involved in late embryogenesis abundant (LEA). The bulk of the Aux/IAA genes found in rice were also found to be expressed when the plant was under drought stress. The research found that potatoes and poplar trees with overexpressed *YUC6* genes have improved drought tolerance and auxin overproduction characteristics [86]. Moreover, under both normal and dry conditions, the interaction between ABA (abscisic acid) and auxin signaling is crucial for regulating root growth and development. The phytohormone abscisic acid (ABA), referred to as the “stress hormone”, alters several morphological, physiological, biochemical, and molecular processes in plant root tissues to regulate a poplar’s drought resistance [87].

### 6.2. Cytokinin

Cytokinins are crucial for the maturation of plant regulatory mechanisms and adaptations to drought stress [88,89]. These hormones play a central role in cell division (cytokinesis) in both roots and shoots. Under drought stress, cytokinins negatively regulate root system acidosis by modifying root structure and metabolism [90]. In *Populus* species, drought stress has been shown to reduce endogenous cytokinin levels, contributing to increased root-to-shoot ratio, which is a typical adaptive response to water limitation [91]. Moreover, cytokinin signaling interacts with other hormonal pathways to influence root architecture in *Populus*, helping in the formation of deeper and more efficient root systems for water uptake. Transgenic studies in other species (e.g., tobacco and rice) that overexpress cytokinin oxidase (CKX) exhibit enhanced root elongation and lateral root formation, a trait also observed in drought-resistant *Populus* genotypes [92,93]. In *Populus tremula × alba*, drought has been found to downregulate genes related to cytokinin biosynthesis and signaling, correlating with observed reductions in shoot growth and delayed leaf senescence [10]. This indicates a key regulatory role of cytokinin in drought adaptation of *Populus*.

### 6.3. Gibberellinses

Gibberellins (GAs) is the name given to the tetracyclic diterpenoids of carboxylic acids. The primary roles of GAs in plants are as growth regulators and as defense mechanisms against abiotic stressors such as drought. GAs maintain their functions in plants throughout their entire lifespan. In both the immature and mature stages of plant development, gibberellins are utilized to enhance tissue growth by increasing the length of cells and the rate of cell division. Furthermore, they enhance the reproductive and vegetative stages of plant life [94]. Since the recognition and verification of several genes and genetic variations involved in crop drought responses, valuable resources have been made available for the breeding of drought-resistant cultivars using both conventional and cutting-edge gene editing and transgenic technologies [95]. *PtoMYB142* regulates gibberellin catabolism in reaction to drought stress by directly connecting to the promoter of *PtoGA2ox4*, a GA2-oxidase gene activated during drought conditions. The CRISPR/Cas9-mediated deletion of *PtoMYB142* diminished drought resistance [96]. Two modules within the photoperiod pathway explain how poplar (*Populus* spp.) buds go into dormancy: GIGANTEA-like genes (GIs) and the circadian oscillator LATE ELONGATED HYPOCOTYL 2 (LHY2) both control the main target for winter dormancy induction, FLOWERING LOCUS T2 (FT2). But under short-day (SD) conditions, changing LHY2 and GIs does not stop growth and bud development entirely; therefore, other regulatory modules must be involved. Poplar has *PtoHY5a*, which is an ortholog of the photomorphogenesis regulatory factor ELONGATED HYPOCOTYL 5 (*HY5*) has the dual effect of boosting *PtoFT2* expression directly and suppressing *LHY2*’s circadian oscillation indirectly. So, *PtoHY5a* prevents SD-induced bud setting and halts development. Therefore, *PtoHY5a* knockdown promotes dormancy induction. Furthermore, *PtoHY5a* controls gibberellic acid (GA) concentrations in apical buds, which inhibits poplar bud-break. Also, following phytochrome *PHYB2*, *PtoHY5a* regulates photoperiodic growth. So, *PtoHY5a* regulates the seasonal growth of poplars by controlling GA levels to regulate bud-break and the *PtoPHYB2-PtoHY5a-PtoFT2* module to establish the start of winter dormancy [97].

### 6.4. Abscisic Acid

Abscisic acid is an essential phytohormone for signaling during drought stress [98]. Our current understanding of the role of expansins in woody plants is limited. The *Populus* and expansin gene family, which is 36 members strong and is subdivided into 4 subfamilies, was the focus of a recent study. They also investigated *Populus tremula* L. expansin genes (*PtEXs*), along with their molecular composition. Additionally, they investigated how phytohormones and abiotic stresses affected the expression of these genes [99]. Additional research indicates that when *PdNF-YB21* is overexpressed in poplar trees, it enhances drought resistance through the root xylem channel development which is both bigger and highly lignified. Conversely, nf-yb21, a poplar mutant generated by CRISPR/Cas9, exhibited stunted root development and reduced drought resilience. *PdNF-YB21* interacted with *PdFUSCA3*, also known as PdFUS3, a transcription factor with a B3 domain. An essential gene involved in abscisic acid (ABA) synthesis, *PdNCED3*, was directly induced by PdFUS3. A dramatic increase in the concentration of ABA (abscisic acid) in the dry roots of poplar trees was a result of this. The co-expression of poplar *NF-YB21* and *FUS3* significantly increased the expression of *PdNCED3*. Additionally, ABA (abscisic acid) improved root development and drought resistance by increasing indoleacetic acid transport to root tips [100].

### 6.5. Salicylic Acid

One of the most important hormones that plants have for dealing with biotic and abiotic stresses and for balancing development and immunity is salicylic acid (SA), which is also called the sixth phytohormone [101]. Methodical identification and exploration of SA biosynthesis are thus crucial to plant science and the development of novel medicines. The model plant Arabidopsis thaliana has been the subject of research in terms of SA biosynthesis [102]. In their study, Xiao et al. utilized a combination of genome-wide association studies (GWASs), metabolite and expression profiling methods, and *Populus* to investigate the genetic architecture of SA biosynthesis. A total of 300 unique *Populus tomentosa* Carr. individuals had nine different SA biosynthesis levels, and initial intermediates were measured [103]. Drought, according to previous studies, increased endogenous JA and ABA (abscisic acid) levels in *Brassica napus*, which in turn increased ABA/SA and (ABA + JA)/SA [104]. There was a drop in reducing potential [NAD(P)H/NAD(P) + and GSH/GSSG] and an increase in reactive oxygen species (ROS) and proline alongside the changes in endogenous hormonal balance. The SA pretreatment effectively removed the excess O2 that had built up due to the drought [104]. Furthermore, salicylic acid levels were five times greater in drought-stricken plants than in normally occurring evergreen *Phillyrea augustifolia* shrubby plants [105].

### 6.6. Ethylene

Researchers discovered that ethylene enhances plants’ resilience to drought conditions [106]. Several developmental processes are regulated by the multifunctional phytohormone ethylene when environmental conditions are unfavorable [107]. Ethylene alters RSA by enhancing auxin production and the machinery that transports it. Multiple investigations have shown that ethylene causes an increase in the expression of auxin efflux genes (*PIN 1*, *2*, and *4*), as well as the inflow gene (*AUX1*) [108]. Ethylene positively regulates poplar’s responses to canker caused by the *hemibiotrophic* fungus *Dothiorella gregaria*. Applying the biosynthetic precursor of ethylene, 1-aminocyclopropane-1-carboxylic acid (ACC), to *Populus tomentosa* significantly enhanced the plant’s disease resistance, along with enhancing H_2_O_2_ accumulation and the expression of the pathogen-related protein (PR) gene. An inhibitor of ethylene production, aminoethoxyvinyl glycine (AVG), was used to reduce disease resistance. *Populus tomentosa* showed improved defenses and disease resistance after overexpressing the *PtoACO7* gene, which is involved in ethylene production. In addition, they demonstrated that the ethylene-induced defensive response, which does not rely on the salicylic acid pathway, requires ROS signaling. In poplars, elevated H_2_O_2_ levels and the production of the NADPH oxidases *PtoRbohD/RbohF* were seen as a result of the overexpression of ACC or PtoACO7. Inhibiting NADPH oxidase decreased PR gene expressions and ethylene-induced disease resistance while treating H_2_O_2_ completely restored AVG-induced disease hypersensitivity. It follows that ethylene contributes to disease resistance through ROS formation and PR gene expression activation. Changing ethylene production or its signaling route could significantly improve disease resistance in woody plants [109].

### 6.7. Brassinosteroids

Plant growth hormones are considered an effective means of mitigating the adverse effects of salt. Brassinosteroids (BRs) are phytohormones that exhibit significant benefits against various abiotic stressors. BRs can alleviate the detrimental effects of salt stress by enhancing water and nutrient absorption, membrane integrity, antioxidant functions, and osmolyte production while preserving hormonal equilibrium [110]. Previous research has shown that brassinosteroids improve drought stress responses in Arabidopsis, wheat, and Brassica species [111]. In Arabidopsis, a bifunctional cytochrome P450 monooxygenase known as *AtCYP90D1* has been studied for its role in the production of brassinolides, namely in the C-23 hydroxylation phase. In this study, the functional characteristics of *PtoCYP90D1*, one of the *AtCYP90D1* homologous genes from *Populus tomentosa*, are revealed. According to the qRT-PCR analysis, *PtoCYP90D1* was found to be highly expressed in both roots and old leaves. In poplar, *PtoCYP90D1-OE* overexpression improved cell layers, xylem area, growth, and biomass yield. Transgenic plants displayed significantly taller and wider stems compared to their wild counterparts. In contrast, CRISPR/Cas9 transgenic plants expressing the *PtoCYP90D1-KO* mutant had much lower biomass production. Following this, studies demonstrated that *PtoCYP90D1-KO* lines did not exhibit a significant increase in cell wall components compared to wild-type plants, while *PtoCYP90D1-OE* lines did. In sum, the data demonstrate that *PtoCYP90D1* has a positive impact on the growth rate and biomass output of poplar trees, which is beneficial for all the agricultural and industrial applications of this versatile plant [112].

### 6.8. Jasmonic Acid

Jasmonic acid (JA) is a lipid-derived phytohormone known for regulating defense mechanisms and improving plant tolerance to abiotic stresses such as drought [113]. JA and its derivatives, collectively called jasmonates (JAs), modulate resource allocation and inhibit root growth under stress conditions [114,115]. In *Populus* species, drought stress induces the upregulation of jasmonic acid biosynthesis genes such as LOX, AOS, and OPR3, which are key components of the JA signaling pathway [77]. This hormonal shift is associated with enhanced expression of stress-related genes and changes in root architecture. Specifically, in *Populus euphratica*, JA signaling has been implicated in root hydrotropism and lateral root development under osmotic stress [116]. The MYC2 transcription factor, central to JA signaling, is also responsive in *Populus trichocarpa* under drought, indicating a conserved role in stress adaptation. Moreover, comparative transcriptomic studies have revealed that JA-responsive genes are differentially expressed in drought-tolerant vs. susceptible *Populus* genotypes, underscoring the hormone’s role in species-specific drought resilience [117].

### 6.9. Peptides

**Figure 4:**
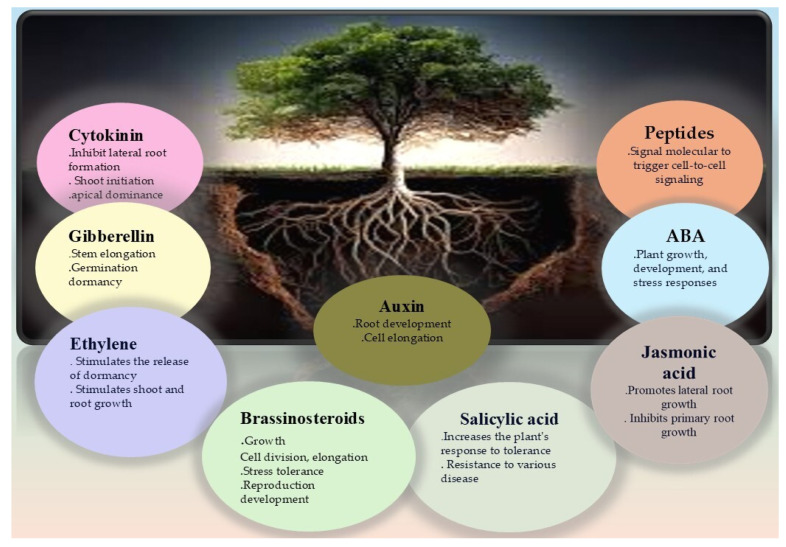
When plants are drought-stricken, phytohormones mediate a cascade of physiological reactions that control root development. Root growth and stomatal closure are both aided by auxins and cytokinin, which in turn promote water uptake, and abscisic acid (ABA) is essential for both processes. Hormones such as ethylene and jasmonic acid have a role in stress signaling and adaptive responses, which allow plants to improve their drought resilience by optimizing their root architecture.

It is evident that safer and more eco-friendly growth regulators are essential for sustainable agro-forestry output. A peptide is a small biological molecule that makes up the plant proteome. Since the initial revelation of signal peptides in plants, a plethora of short peptides (sPEPs, 2–100 amino acid residues) encoded by tiny open reading frames (sORFs) have been found. Plant development, biotic response, signal transduction, and growth are all regulated by these peptides [118]. However, whether or not these peptides facilitate drought-related long-distance signaling remains unclear. An important peptide for shoot apical meristem growth is *CLAVATA3* (*CLV3*), which has been the subject of much research in plants. In land plants, the phytohormone abscisic acid controls stomatal motility to prevent water loss. However, no signaling molecules that can facilitate the accumulation of abscisic acid in leaves have been identified thus far [119]. Researchers have found that the *CLE25* peptide sends signals through vascular tissues to control transpiration in Arabidopsis, which in turn affects abscisic acid generation and stomatal regulation. The root’s resistance to drought stress was enhanced by the expression of the peptide-related gene in the vascular tissues. The peptides travel up the plant’s stems to its leaves, where they regulate the abscisic acid accumulation and trigger stomatal closure, making the plant more resistant to drought stress [120]. Additional peptides include phytosulfokine (PSK), linked to cell proliferation; LUREs, which direct pollen tube growth; RALF, which modulates root development; STOMAGEN, associated with stomatal development; and CIF, related to the formation of the Casparian strip diffusion barrier. A significant peptide associated with salinity and drought stress has recently been identified by researchers—*AtPep3* [120]. To improve poplar tree management and breeding programs to boost their drought resilience in forestry and agriculture, it is vital to understand these hormonal changes, as will be shown in Figure 4.

## 7. Plant Growth-Promoting Rhizobacteria Improve Plant Health

To reduce stress, Rhizobacteria and mycorrhizae stimulate phytohormone, siderophore, antioxidants, phosphate solubilization, and ethylene reduction. They are used in solid, liquid, metabolite, or polymeric forms depending on crop needs [121]. Drought stress affects plant morphology, energy metabolism, signaling pathways, reactive oxygen species generation, and hormone synthesis. However, several attempts to overcome this impediment were unsuccessful and only temporarily effective. Plant growth-promoting rhizobacteria (PGPR) generates indole acetic acid and gibberellins. PGPR produced active enzymes under drought and waterlogged conditions. This technology enhances plant growth and crop yield while being ecologically friendly [122]. Various tissue types have been identified as colonizing trees [123] and nodes, foliage, and stalks [124]. Invasive fungi can be found in wood, bark, and other organic materials [125]. Many distinct dark septate endophytes have been identified in plant roots from different parts of the globe (DSE) [126]. Each isolate was tested for nitrogen fixation, phosphate solubilization/mineralization, IAA, and siderophore synthesis [127].

**Figure 5:**
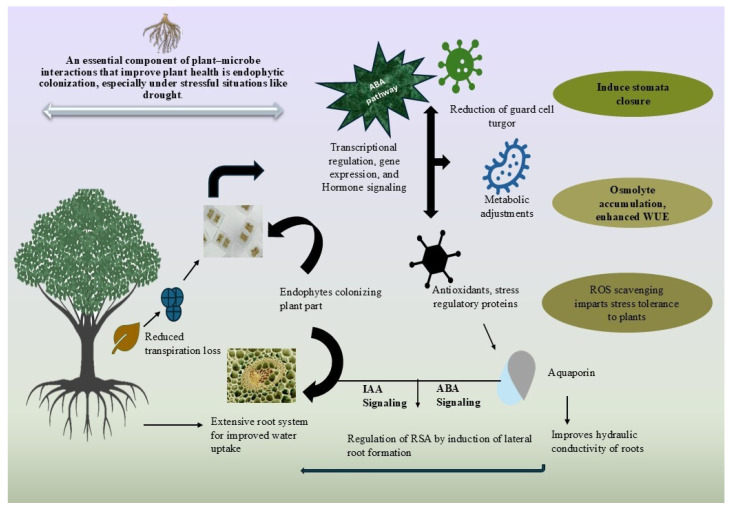
Endophytic colonization involves beneficial microbes that reside inside plant tissues, promoting growth and stress tolerance. These microbes can help plants overcome drought by enhancing nutrient uptake, inducing stress resistance pathways, and improving water use efficiency. Ultimately, endophytic colonization contributes to improved plant health and survival in challenging environments like drought.

Whether useful or harmful, microbes can infect plants and have the rhizosphere competence to enter and multiply in plants and be transferred to other hosts. Endophyte colonization depends on plant tissue type, genotype, microbial taxon and strain, and biotic and abiotic environmental situations [128]. The inoculum density, age, and species of the plant, growing medium, fungal genus, and rate of conidia application considerations affect endophytic colonization [129]. JA controls root endophyte density [130]. Many investigations have shown that *Enterobacter* sp. does not encode cellulose-degrading proteins, consistent with its nonpathogenic behavior during endophyte–poplar tree contact [131]. Research indicates that the influence of rhizobacteria strains JS and charcoal mineral fertilizer on biomass yield and physiological characteristics is beneficial for cultivating poplar trees in marginal soils, such as reclaimed land, and promotes wood pellet utilization by enhancing soil quality [132]. Endophytic colonization is a vital component of plant–microbe interactions that improve plant health, especially under challenging conditions such as drought, as shown in Figure 5.

## 8. Transgenic Approaches

Improving plant productivity, quality, and tolerance to abiotic and biotic stress can be achieved by plant genetic transformation [133]. Various established genetic transformation techniques can reliably incorporate additional genes into the nuclear genomes of diverse plant species. Despite decades of technical progress, effective plant transformation and regeneration continue to pose challenges for numerous species [134]. Transgenic plant regeneration and biomolecule delivery are the two primary processes in plant genetic engineering. The process of biomolecules penetrating plant cells and then regenerating transgenic plants from in vitro grown explants, whether by de novo organogenesis or somatic embryogenesis, is the primary obstacle to successful plant genetic transformation [135]. In addition, from the uses already mentioned, there is a lot of room for improvement when it comes to figuring *Populus* species with new and improved traits [136]. Multiple *Populus* species have been genetically modified by *Agrobacterium tumefaciens* [137]. The study illustrates the durable genetic change in two poplar species, *Populus* angustifolia and *Populus* balsamifera, mediated by *Agrobacterium tumefaciens*. The binary vector *pCAMBIA*-Npro-long-Luc contains the luciferase reporter gene [138]. Genes linked to drought response, including the *AREB1* gene, which controls ABA (abscisic acid) signaling, can be precisely edited thanks to this technology. Research has indicated that when drought circumstances are present, CRISPR-modified plants have enhanced physiological characteristics [139]. Several research has shown how CRISPR-based genetic alteration can improve the quality of wood [140,141]. In recent years, CRISPR/Cas9 genome editing technologies have been utilized in popular plants [142]. The efficacy of Cas12a in woody tree species remains unknown. The hybrid poplar (*Populus alba × Populus glandulosa*) clone 84 K is utilized to evaluate specific alterations through the CRISPR/Cas12a system [143,144].

## 9. Discussion

The present analysis highlights the central role of hormonal regulation in enabling *Populus* species to cope with drought stress. *Populus*, as a model genus for woody plants and a key component of many forest ecosystems, exhibits complex and dynamic responses to water deficiency. These responses are primarily driven by intricate hormonal signaling pathways that modulate root architecture, stomatal regulation, and metabolic adaptation under drought conditions.

Multiple phytohormones, including auxin, cytokinin, gibberellins (GAs), abscisic acid (ABA), salicylic acid (SA), ethylene, brassinosteroids (BRs), jasmonic acid (JA), and peptide hormones, play significant roles in drought adaptation in *Populus*. Among them, ABA is the most extensively studied, known for inducing stomatal closure to minimize transpirational water loss and maintain internal water balance. This regulation is particularly critical for *Populus*, as it often grows in environments where rapid adjustments to water availability are essential for survival.

Root system plasticity is another hallmark of drought adaptation in *Populus*. Under drought conditions, *Populus* species alter their root morphology by extending deeper taproots and enhancing lateral root development to access deeper soil moisture. Auxins promote these changes by stimulating lateral root formation and elongation, thus increasing the root system’s capacity to absorb water even in arid soils. This adaptive strategy often compensates for reduced aboveground growth due to water limitation.

Cytokinins, known for regulating shoot and root development, help delay leaf senescence and promote root initiation under drought stress conditions in *Populus*. GAs, although generally growth-promoting, are often downregulated during drought to reduce elongation and conserve energy. SA and BRs contribute to stress mitigation by enhancing antioxidant enzyme activities, improving photosynthetic performance, and regulating stress-responsive gene expression. JA, while more traditionally associated with defense mechanisms, also influences root system architecture and drought-responsive signaling. Recent findings indicate that peptide hormones serve as intercellular messengers, enhancing the coordination of stress responses across various tissues in *Populus*.

In addition to hormonal modulation, *Populus* undergoes several physiological and structural changes to withstand drought. Metabolically, drought stress induces the accumulation of osmoprotectants like proline and soluble sugars, which help stabilize cellular structures and mitigate oxidative damage caused by reactive oxygen species (ROS). These ROS, primarily produced in organelles such as chloroplasts, mitochondria, and peroxisomes, can damage membranes and proteins if not regulated. The hormone-mediated upregulation of antioxidant enzymes—such as superoxide dismutase (SOD), catalase (CAT), and peroxidases—plays a vital role in *Populus*’ ability to counteract oxidative stress.

Structural adjustments at the root–soil interface are equally crucial. *Populus* species frequently modify their root anatomy, enhancing hydraulic conductivity while minimizing vulnerability to cavitation. Symbiotic relationships with mycorrhizal fungi further improve drought resistance by increasing water and nutrient uptake. Such associations are particularly beneficial to *Populus*, given its fast-growing nature and high water demand.

Collectively, these hormonal and structural responses underscore the adaptive resilience of *Populus* under drought stress. The integration of signaling pathways, root system plasticity, and protective metabolic processes allows *Populus* to maintain functional integrity and resource acquisition during water deficit conditions.

## 10. Conclusions

Understanding the hormonal regulation of drought stress responses is essential for enhancing the resilience of *Populus* species, which are particularly vulnerable to water limitations. As drought becomes increasingly frequent and severe due to climate change, improving the drought tolerance of *Populus* is crucial for sustainable forestry, biomass production, and ecosystem health.

Future research should focus on unraveling the molecular mechanisms through which phytohormones influence drought adaptation specifically in *Populus*. Special attention must be given to the genetic networks that mediate hormone signaling in response to environmental stimuli. The use of advanced omics technologies such as genomics, transcriptomics, proteomics, and metabolomics will be instrumental in identifying key genes and pathways involved in hormonal regulation during drought stress in *Populus*.

Breeding and biotechnological strategies for drought-resilient *Populus* varieties are another vital avenue. Approaches such as marker-assisted selection, genetic transformation, and genome editing tools like CRISPR-Cas9 can be employed to improve root traits, enhance hormone signaling pathways, and boost overall stress tolerance. Identifying genetic markers linked to favorable drought response traits in *Populus* will accelerate the selection and development of robust genotypes.

It is also essential to validate laboratory findings under field conditions to assess the performance of hormone-regulated responses in real-world environments. Long-term trials will help determine the practical utility of these traits in various *Populus* genotypes and guide future forest management practices.

Given the escalating global issue of water scarcity, advancing our understanding of how *Populus* species respond to drought at the physiological, molecular, and ecological levels is critical. An interdisciplinary approach involving ecologists, plant physiologists, geneticists, and forestry experts will be key to developing holistic strategies that enhance *Populus* resilience while maintaining ecosystem sustainability. Strengthening these adaptive mechanisms will not only secure *Populus* productivity but also contribute to broader efforts in climate change mitigation and sustainable land use.

## Figures and Tables

**Table 1:** Phytohormones function to protect plants from drought stress.

Hormones	Stress Resilience Mechanism	Mode of Action	Species	References
Auxin	Facilitates root elongation and promotes lateral root development.	Facilitates root development and enhances water absorption.	*Populus alba*	[72]
Cytokinins	Preserves shoot meristem functionality during stress.	Modulates nutrition distribution, postpones aging.	*Populus tremula*	[73]
Gibberellins	Regulates growth to optimize water conservation.	Inhibits stem elongation under drought conditions.	*Populus deltoides*	[74]
Abscisic acid (ABA)	Modulates stomatal closure to minimize water loss.	Stimulates the expression of stress-responsive genes.	*Populus trichocarpa*	[75]
Salicylic acid (SA)	Improves antioxidant defense mechanisms.	Reduces the oxidative damage.	*Populus euphratica*	[76]
Ethylene	Regulates root structure in water-scarce environments.	Facilitates the development of adventitious roots.	*Populus nigra*	[77]
Brassinosteroids	Enhances photosynthetic efficacy under stress conditions.	Stabilizes membranes and modulates reactive oxygen species levels.	*Populus cathayana*	[78]
Jasmonic acid (JA)	Enhances structural resilience against desiccation.	Induces stress-related protein synthesis.	*Populus simonii*	[79]
Peptides	Improves signals for stress adaptation.	Regulates metabolic and hormonal pathways.	*Populus tomentosa*	[80]

## Data Availability

Not applicable.

## Abbreviations

The following abbreviations are used in this manuscript:

ABA	Abscisic acid
IAA	Indole-3-acetic acid (a form of auxin)
CKX	Cytokinin oxidase
GA or GAs	Gibberellins
SA	Salicylic acid
JA	Jasmonic acid
JAs	Jasmonates (includes JA and derivatives)
LOX	Lipoxygenase
AOS	Allene oxide synthase
OPR3	12-oxophytodienoate reductase 3
MYC2	(Not an acronym; it is a transcription factor involved in JA signaling)
CK	Cytokinin
ROS	Reactive oxygen species
CAT	Catalase
SOD	Superoxide dismutase
APX	Ascorbate peroxidase
GPX	Glutathione peroxidase
PRX	Peroxiredoxin
GSH	Glutathione
AMF	Arbuscular Mycorrhizal Fungi
PGPR	Plant growth-promoting rhizobacteria
H_2_O_2_	Hydrogen peroxide
RSL4	Root hair defective six-like 4 (a transcription factor involved in root hair development)
ARR1/ARR12	Arabidopsis response regulators (involved in cytokinin signaling)
FT2	FLOWERING LOCUS T2
HY5	ELONGATED HYPOCOTYL 5 (photomorphogenesis regulatory factor)
CRISPR/Cas9	Clustered regularly interspaced short palindromic repeats/CRISPR-associated protein 9
LEA	Late embryogenesis abundant (genes associated with stress tolerance)
JAZ	Jasmonate ZIM-domain (repressor proteins in JA signaling)
COI1	Coronatine-insensitive 1 (receptor in JA pathway)
JAUP1	Jasmonic acid upregulated protein 1
DSE	Dark septate endophytes
BRs	Brassinosteroids
EPF	Epidermal patterning factor
CK	Cytokinin
GA2ox4	Gibberellin 2-oxidase 4 (enzyme involved in GA catabolism)
